# Inhibitory projections from the ventral nucleus of the trapezoid body to the medial nucleus of the trapezoid body in the mouse

**DOI:** 10.3389/fncir.2014.00083

**Published:** 2014-07-29

**Authors:** Otto Albrecht, Anna Dondzillo, Florian Mayer, John A. Thompson, Achim Klug

**Affiliations:** ^1^Department of Physiology and Biophysics, School of Medicine, University of ColoradoAurora, CO, USA; ^2^Department of Neurosurgery, School of Medicine, University of ColoradoAurora, CO, USA

**Keywords:** VNTB, MNTB, glycinergic, auditory, calyx of held, trapezoid body, inhibition

## Abstract

Neurons in the medial nucleus of the trapezoid body (MNTB) receive prominent excitatory input through the calyx of Held, a giant synapse that produces large and fast excitatory currents. MNTB neurons also receive inhibitory glycinergic inputs that are also large and fast, and match the calyceal excitation in terms of synaptic strength. GABAergic inputs provide additional inhibition to MNTB neurons. Inhibitory inputs to MNTB modify spiking of MNTB neurons both *in-vitro* and *in-vivo*, underscoring their importance. Surprisingly, the origin of the inhibitory inputs to MNTB has not been shown conclusively. We performed retrograde tracing, anterograde tracing, immunohistochemical experiments, and electrophysiological recordings to address this question. The results support the ventral nucleus of the trapezoid body (VNTB) as at least one major source of glycinergic input to MNTB. VNTB fibers enter the ipsilateral MNTB, travel along MNTB principal neurons and produce several bouton-like presynaptic terminals. Further, the contribution of GABA to the total inhibition declines during development, resulting in only a very minor fraction of GABAergic inhibition in adulthood, which is matched in time by a reduction in expression of a GABA synthetic enzyme in VNTB principal neurons.

## Introduction

The medial nucleus of the trapezoid body (MNTB) is an auditory brainstem nucleus located in the ventro-medial aspect of the brainstem. It consists of about 3000–5000 principal neurons (Rodríguez-Contreras et al., 2006; Kulesza, 2008) that receive neural excitation from globular bushy cells located in the contralateral antero-ventral cochlear nucleus (AVCN; Held, 1893; Morest, 1968). Almost all excitatory input to MNTB neurons is conveyed via the giant calyx of Held synapse (Held, 1893; Guinan and Li, 1990). Evidence also exists for additional non-calyceal inputs from an unknown source (Banks and Smith, 1992; Hamann et al., 2003). Each MNTB neuron receives excitatory input from exactly one calyx (Rodríguez-Contreras et al., 2006), which produces large and fast excitatory currents (Forsythe, 1994; Taschenberger and von Gersdorff, 2000). The results of many studies have provided a good understanding of the excitatory input (von Gersdorff and Borst, 2002; Schneggenburger and Forsythe, 2006; Borst and van Hoeve, 2012).

Much less attention has been paid to inhibitory inputs to MNTB neurons. The inhibitory inputs comprise both GABAergic and glycinergic components, although the contribution of GABA declines during development, resulting in little GABAergic inhibition in the adult (Awatramani et al., 2005). However, at all stages, glycinergic inhibition to MNTB is large and fast, and able to follow rapid stimulus trains (~several 100 Hz; Awatramani et al., 2004). The synaptic conductance of the inhibitory input matches the extremely large synaptic conductance produced by the calyceal excitatory input, and is able to suppress MNTB spiking when activated (Awatramani et al., 2004). Even after prolonged stimulation, the inhibitory input continues to produce large, fast, and phasic synaptic currents (Florian Mayer, unpublished data). *In-vivo*, neural inhibition to MNTB shapes its response to sound (Green and Sanes, 2005; Kopp-Scheinpflug et al., 2008; Tolnai et al., 2008). Further, blocking glycinergic inhibition *in-vivo* reveals that some inhibitory inputs shape responses at or near the neurons' best excitatory frequencies, while others act off best frequency (Kopp-Scheinpflug et al., 2008).

Despite the importance of glycinergic inhibition for information processing in the MNTB, there are gaps in our knowledge about the origin of the inhibitory inputs. Evidence exists for both the ventral nucleus of the trapezoid body (VNTB) (Kuwabara et al., 1991; Thompson and Schofield, 2000) and MNTB itself (Guinan and Li, 1990; Kuwabara and Zook, 1991) as putative sources.

Our goal was to determine the source(s) of glycinergic inhibition to MNTB. We used a transgenic mouse line in which glycinergic neurons express green fluorescent protein (GlyT2-GFP mouse; Zeilhofer et al., 2005), and performed retrograde and anterograde tracing studies, immunohistochemistry, and electrophysiology. Our findings suggest that the VNTB is one area that sends substantial glycinergic inputs to MNTB. VNTB fibers travel along MNTB principal neurons and produce several bouton-like presynaptic terminals. The developmental shift in GABAergic contribution is matched by reduced detection of the GABAergic marker, GAD67, in VNTB principal neurons.

## Methods

All experimental procedures complied with all applicable laws and NIH guidelines and were approved by the University of Colorado IACUC. All experiments were conducted in a transgenic GlyT2-GFP mouse line, in which glycinergic neurons are labeled with enhanced green fluorescent protein [eGFP; Poyatos et al., 1997; Zeilhofer et al., 2005; JAX registry code: Tg(Slc6a5-EGFP)1Uze].

### Retrograde tracing

**Figure 1:**
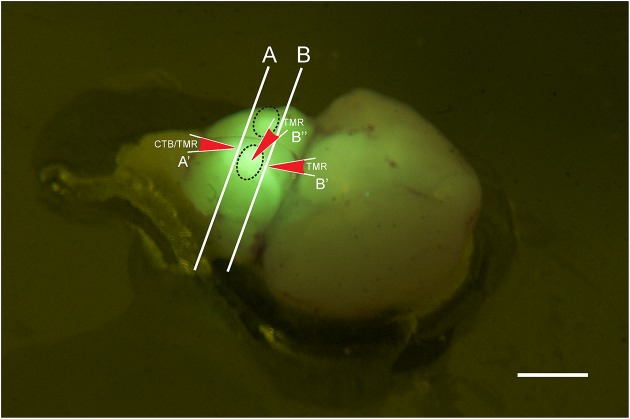
**Photo of a brain stem explant of a young (p2) GlyT2-GFP mouse showing the ”bulbs” on the ventral surface of the explant (dashed oval lines).** The image was taken through a yellow filter while the brain explant was illuminated with a 405 nm laser. With this type of lighting, the bulbs light up brightly and can easily be distinguished, presumably because they contain the VNTB with GFP positive neurons close to the brain surface, and presumably because other major sources of GlyT2-GFP label, such as the MNTB, are located close by. The locations of the two different coronal cutting planes for retrograde and anterograde injections are shown by lines A and B, respectively, and the injection sites and their directionality are depicted by the red arrows A', B', and B.” Scale bar: 1 mm.

GlyT2-GFP mice aged p14 to p138 were anesthetized with pentobarbital (120 mg/kg bodyweight) and perfused transcardially with ice-cold phosphate buffered saline (PBS; NaCl: 137 mM, KCl: 2.7 mM, KH_2_PO_4_: 1.76 mM, Na_2_HPO_4_: 10 mM; all chemicals from Sigma-Aldrich). After exsanguination and perfusion, the animals were decapitated and the brain removed. Brain explants were cut along the caudal border (line “A” in Figure 1) of two ventral prominent “bulbs” containing the VNTB (black dotted lines in Figure 1), exposing the caudal end of the MNTB in the coronal cutting plane (white line “A” in Figure 1). Brainstem explants were placed in oxygenated (95% O_2_, 5% CO_2_) dissecting solution (NaCl: 125 mM, KCl: 2.5 mM, MgCl_2_: 1 mM, CaCl_2_: 0.1 mM, glucose: 25 mM, NaH_2_PO_4_: 1.25 mM, NaHCO_3_: 25 mM, ascorbic acid: 0.4 mM, myo-inositol: 3 mM, pyruvic acid: 2 mM). Injection pipettes were pulled from borosilicate glass (Harvard Apparatus; GC150F-10) using a Zeitz DMZ Universal Puller (Zeitz Instruments, Germany) and filled with PBS containing 1.0 mg/ml solution of cholera toxin subunit-b conjugated to Alexa 555 (*n* = 11) (Molecular Probes; C-34776) (Conte et al., 2008, 2009), or dextran tetramethylrhodamine 555 (*n* = 7) (TMR; 3000 mW; 1%-dilution in 0.9% saline; Molecular Probes; D-7162). Pipette resistances varied from 2.5 to 3.5 MOhm.

Tracer injection locations were confirmed visually by inspecting the exposed caudal end of the explant (for further anatomical reference, see Franklin and Paxinos (2008); Franklin and Paxinos, Plate 78, Bregma ~ −5.7 mm). Based on this, the injection site was within the area marked as “Tz” (nucleus of the trapezoid body = MNTB). Electrodes were inserted into the tissue block in a perpendicular direction, as indicated by the red arrow labeled A' (Figure 1).

The injections consisted of 2–10 pressure pulses (15 psi, 50 ms), directed perpendicularly at the MNTB in the coronal plane of the brainstem. Single pressure pulses were administered at intervals of 10–15 s to allow for the dye to spread. For the TMR injections, additional electrical stimulation was performed to enhance the dye uptake through electroporation (Burger et al., 2005; Ford et al., 2009) After the injections, brainstems were incubated in oxygenated artificial cerebrospinal fluid (ACSF; NaCl: 125 mM, KCl: 2.5 mM, MgCl_2_: 1 mM, CaCl_2_: 2 mM, glucose: 25 mM, NaH_2_PO_4_: 1.25 mM, NaHCO_3_: 25 mM, ascorbic acid: 0.4 mM, myo-inositol: 3 mM, pyruvic acid: 2 mM) for 1–4 h. Subsequently, the brainstems were post fixed in 4% paraformaldehyde (PFA) overnight. On the next day the brainstems were washed three times in PBS (5 min for each washing step), covered in 4% agar and then cut into 50–80 μm thick slices on a Leica VT1000S vibratome. Each slice was mounted using Fluromount-G (Southern Biotech; Cat.-No: 0100-01) and coverslipped. A subset of brains (*n* = 7) were additionally labeled with fluorescent blue Nissl (1:100 dilution; Invitrogen; N21479).

### Anterograde tracing

The preparation was similar as for retrograde tracing, except that explanted brains were either not cut, or cut rostral to MNTB (Line “B” in Figure 1; compare with Bregma −4.6 mm; Franklin and Paxinos, 2008). TMR was injected directly from the ventral side of the brainstem into one of the prominent bulbs containing the VNTB (oval structures in Figure 1, injection site and direction is labeled by red arrow B”) or into the exposed rostral end of VNTB (red arrow B', Figure 1). This location corresponded to Bregma ~ −4.6 mm;(Franklin and Paxinos, 2008), Plate 69 [labeled “MVPO” (medioventral periolivary nucleus) = VNTB].

In addition to the pressure pulses, a series of square voltage steps (8 TTL pulses, 8 Volts, 50 ms duration, 50 ms interpulse interval) were applied (Burger et al., 2005; Ford et al., 2009). The stimulus train was usually repeated 10–20 times after each pressure injection pulse to enhance the dye uptake via electroporation. The stimulus train was programmed in MC Stimulus software and used to control a STG-1002 stimulator (both Multi Channel Systems, Germany). The output of the stimulator was connected to a stimulation isolation unit (Iso-Flex, A.M.P.I., Israel), which was used to amplify the voltage steps to 55 V. Following the injection and stimulation, brainstem explants were treated as for retrograde tracer injections (incubation, fixation, and wash cycles). Then, brainstems were cut into sections of 200–500 μm, and cleared (Clear^T2^, Kuwajima et al., 2013). After the last incubation step, slices were mounted with custom-made spacers, coverslipped with the same clearing solution that was used to incubate the sections [a mix of 50% formamide and 20% poly(ethanyl glycol), 8000 mW], and imaged.

### Immunohistochemistry

For immunostaining of the brain tissue against GAD67 (glutamic acid decarboxylase-67 kDa) GlyT2-GFP mice aged p14 (6 animals), p59 (3 animals), and p70 (2 animals) were perfused transcardially with PBS and 4% PFA. For the antibody labeling against GlyT2, five adult mice (two wild type animals aged p51 and 3 GlyT2-GFP mice, aged p59, p60, and p61) were sacrificed. Their brainstems were taken out and post fixed in 4% PFA for 2 h. After being washed in PBS (3 × 5 min), the brainstems were covered in 4% agarose and cut into 40 μm thick slices. These slices were washed 3 × 10 min in PBS and incubated in AB media [0.1 M phosphate buffer (PB; KH_2_PO_4_: 50 mM, Na_2_HPO_4_: 150 mM], 150 mM NaCl, 3 mM Triton-X, 1% bovine serum albumin; 1% normal goat serum) containing unlabeled Fab fragments of goat anti-mouse IgG (Jackson ImmunoResearch) overnight to enhance the specificity of the primary antibody in mouse tissue (in the case of the GlyT2 labeling, the Fab fragments were left out). After this blocking step the slices were kept in the primary antibody solution for 2 days at 4°C. The primary antibodies were a purified mouse monoclonal IgG2a against GAD67 (Millipore; MAB5406; RRID: AB_2278725) and a guinea pig polyclonal antibody raised against a rat GlyT2 C-terminal region (Millipore; AB1773; RRID: AB_90953). Specificity of both antibodies has been shown previously (Ito et al., 2007; Dufour et al., 2010). Following the incubation in the primary antibody solution, the slices were washed 3 × 10 min in PBS and incubated in secondary antibody solution for 2 h. The secondary antibodies were acquired from Invitrogen (goat anti-mouse IgG conjugated with Alexa 568; A11031; RRID: AB_144696 and goat anti-guinea pig IgG conjugated with Alexa 568; A11075; RRID: AB_141954). After another three washing steps in PBS, the slices were briefly washed in PB, mounted on glass slides using Fluromount-G (SouthernBiotech, Cat.-No.: 0100-01) and coverslipped.

### Imaging

The retrograde and anterograde tracer-injected brainstem slices were imaged on three different systems: 3I Vivo (Denver, USA) equipped with a 20×/N.A. 1.0 DIC water objective (1.0 mm working distance) and Slidebook 5.5 imaging software, Olympus Fluoview with 10×/N.A. 0.4, 20× oil /N.A. 0.8 and 60× oil /N.A. 1.4 objectives using Fluoview imaging software and Olympus FV1000 FCS/RICS with 10×/N.A. 0.4, 20×/N.A. 0.75 and 60× water /N.A. 1.2. Immunolabeled brain slices were imaged with the Olympus Fluoview system only. Images were background-corrected either online using the respective imaging software or *post-hoc* in ImageJ.

### Image analysis

#### VNTB delineation

**Figure 2:**
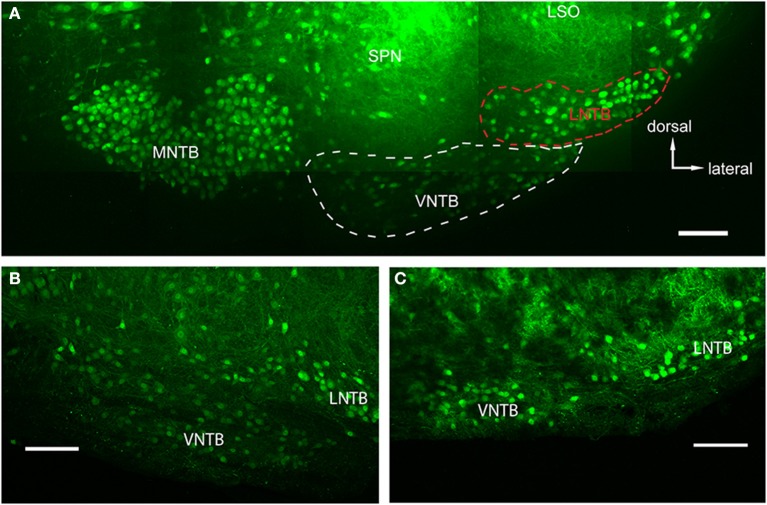
**Glycinergic auditory nuclei in a brainstem of a p14 GlyT2-GFP mouse. (A)** Maximum projection of 10 tiled confocal stacks through a 200 μm brain section cleared prior to imaging using the Clear^T2^-protocol. Five major nuclei with glycine label can be seen: the medial nucleus of the trapezoid body (MNTB), the ventral and lateral nuclei of the trapezoid body (VNTB and LNTB, outlined with white and red dashed lines, respectively), the SPN, and the ventral, high-frequency region of the lateral superior olive (LSO). **(B,C)** Closeup of the VNTB/LNTB area, showing the anatomical features used to discriminate between the VNTB and the LNTB. The image in **(B)** represents a more posterior location than **(A)** and **(C)** is located even more posterior than the ones in **(A,B)**. Note the increasing gap between the two nuclei void of glycinergic cells. Scale bars: 100 μm.

We used the GlyT2-GFP signal (i.e., the glycinergic subpopulation of VNTB cells) to delineate the borders of the VNTB (white dotted line in Figure 2A). With this method, the VNTB typically consists of small, stellate or slightly elongated (spindle-like) GlyT2-GFP positive cells, and extends along the ventral border of a coronal brainstem section of the superior olivary complex (SOC). This delineation is consistent with Kuwabara et al. (1991) in terms of VNTB cell shapes, and is consistent with Helfert et al. (1989) in terms of the presence of glycinergic neurons in this nucleus. We defined the medio-lateral extent of VNTB between the lateral border of the ipsilateral MNTB and approximately the medial border of the ipsilateral LSO, consistent with Franklin and Paxinos (2008) and Ollo and Schwartz (1979). In the mouse, the superior paraolivary nucleus (SPN) is located directly above VNTB, marking its dorsal border. SPN was determined by its few and large glycinergic cells (Helfert et al., 1989; Saint Marie and Baker, 1990) and a dense web of glycinergic inputs appearing as a solid green area in our GlyT2-GFP mouse line. Directly lateral to the VNTB lies the lateral nucleus of the trapezoid body (LNTB, red dotted line in Figure 2A), a “comma-shaped” nucleus wrapping around the ventro-lateral border of the LSO. LNTB contains a similar number of glycinergic neurons as the VNTB, but some of them are larger, and rounder, and show brighter fluorescence (Figure 2). We typically observed a distinct non-fluorescent gap between the VNTB and the LNTB; the gap was more prominent in more posterior sections (Figure 2C). Based on these criteria, we were able to distinguish between the two nuclei.

#### Co-localization analysis

For the co-localization analysis of the GABAergic marker GAD67 in glycinergic (GFP+) VNTB neurons we chose the automatic co-localization algorithm developed by Li et al. (2004) that is implemented as one of ImageJ's plugins (Just Another Co-Localization Plugin; JACoP). Glycinergic (GFP+) VNTB neurons were marked and cropped out of the stack (in x, y, and z directions) using ImageJ's region of interest (ROI) manager and then analyzed with JACoP. This analysis algorithm is based on a user selecting a square area containing one glycinergic VNTB neuron from a single image of a confocal stack, and determining the brightness of GAD67 label within this analysis area. The result is subsequently correlated with the GFP signal intensity in the same area on a pixel-by-pixel basis. The amount of co-variation of signal intensities in both channels is formulated as the ICQ (intensity correlation quotient), with higher ICQ values indicating a higher degree of co-localization. The co-localization color map (after Jaskolski et al., 2005) was used to look for co-localization hot spots in young and adult animals. The statistical analysis was done in MS Excel and Sigmastat.

#### Semi-automated injection site quantification

Epifluorescent images of brain slices (80 μm thick) containing injected MNTB were collected using an Olympus BX 41 microscope (obj. × 10, N.A. 0.3) with TRITC (for tracer fluorescence) and DAPI (Nissl fluorescence) filter cubes. Image dimensions were 2048 × 2048 pixels with a pixel size of 0.742 μm (x and y) and a 12 bit depth converted to 8 bit for Matlab analysis. The exposure time for the TRITC channel was selected based on the brain section closest to the epicenter of the injection (brightest area) and was kept constant for all images per brain. The collected images were then used for a quantification of the injection site by a custom written routine in Matlab 2013a using base Matlab and the Image Processing Toolbox (Mathworks, Natick, MA). The quantification procedure performed in a custom Matlab GUI consisted of the following steps: (a) the experimenter traced a ROI around the MNTB in the Nissl channel (**Figure 5B**); (b) then the background of the injection signal was objectively averaged in the TRITC channel by selecting three different regions (each sample box in **Figure 5B** was comprised of an area of 1% of the total number of pixels) within the image to capture the distribution of the background signal; (c) calculation of the threshold (see below); (d) counting and displaying all pixels above the threshold located within the MNTB ROI (pink dots in **Figure 5B**). The calculation of the threshold and the number of pixels above threshold was objective and fully automated. This procedure was repeated for each section, resulting in at least 70% of the entire MNTB in the antero-posterior direction being analyzed. Additionally, to estimate the potential amount of injection outside the boundary of the MNTB ROI, a dynamically expanding box ROI was implemented. Initially the box was set to an area 25% greater than the MNTB ROI area and centered on the MNTB ROI. To ensure that the accurate estimation of area of tracer spillover was captured, an algorithm was implemented to detect whether 10% of the total pixels within any side (which varied in length, but were always 1 pixel wide) of the box ROI exceeded threshold. The algorithm would independently expand the sides of the box until all sides contained pixel populations that fell below 10% of threshold. When the parameters of the algorithm were satisfied, the area of pixels that exceeded threshold outside the MNTB and inside the box was calculated to objectively quantify the area of injection tracer spillover.

Data obtained from the custom Matlab GUI were the surface area of the MNTB and the surface area of the injection within the MNTB (in pixels) and were recalculated into volumes. We represent injection accuracy in volume percentages. Threshold was defined as a mean calculated from the MNTB ROI and the three squares selected in the TRITC channel of an image plus two standard deviations (SD), (Threshold = mean + 2^*^SD). All pixels above the threshold within the MNTB ROI were counted as labeled.

If the variability in pixel intensity was high such that the threshold equation resulted in intensities higher than 255, we implemented an upper boundary on the threshold. The second threshold was defined as the maximum intensity minus two standard deviations as calculated from the equation [Threshold_(upper)_ = 255 − 2^*^SD].

The Matlab script for this custom programmed GUI is available for download at the following URL: https://github.com/neuropil/DyeDist).

### Electrophysiology

#### Slice preparation

Slices of brainstem were prepared from mice of either sex ranging in age from p14 to p70. Animals were anesthetized by isoflurane inhalation (IsoFlo, Abbott Laboratories, USA) and decapitated. The brainstem was dissected out and cut into slices of 180 μm with a vibratome (VT1000S, Leica, Wetzlar, Germany) under ice-cold dissection ringer (125 mM NaCl, 2.5 mM KCl, 1 mM MgCl_2_, 0.1 mM CaCl_2_, 25 mM glucose, 1.25 mM NaH_2_PO_4_, 25 mM NaHCO_3_, 0.4 mM ascorbic acid, 3 mM myo-inositol, 2 mM pyruvic acid; all chemicals from Sigma–Aldrich, MO) bubbled for at least 15 min with 5% CO_2_–95% O_2_. Slices were transferred to an incubation chamber containing ACSF (125 mM NaCl, 2.5 mM KCl, 1 mM MgCl_2_, 2 mM CaCl_2_, 25 mM glucose, 1.25 mM NaH_2_PO_4_, 25 mM NaHCO_3_, 0.4 mM ascorbic acid, 3 mM myo-inositol, 2 mM pyruvic acid; all chemicals from Sigma–Aldrich) and bubbled with 5% CO_2_–95% O_2_. Slices were incubated for 1 h at 37°C, after which the chamber was brought to room temperature. Recordings were obtained within 4–5 h of slicing.

#### Whole cell recordings

After incubation, slices were transferred to the recording chamber and continuously perfused with heated and oxygenated ACSF at 2–3 ml/min through a gravity-fed perfusion system. All recordings were performed at 35.5–37°C, controlled by a microcomputer thermometer with a thermo coupling wire that was attached to the 40× water immersion objective (BAT-7001H, Physitemp Instruments, USA). This configuration allowed for measurement of the ACSF temperature within 1–2 mm of the recording site. MNTB neurons were viewed and identified through a Zeiss Axioskop 2 FS plus microscope equipped with Dodt optics and a 40× water-immersion objective (Zeiss, Oberkochen, Germany). Whole cell recordings were performed with an EPC 10 double amplifier (HEKA Instruments, Lambrecht/Pfalz, Germany). Signals were filtered at 5–10 kHz and subsequently digitized at 30–50 kHz using Patchmaster Version 2.40 software (HEKA). Patch pipettes (2.4–3.2 MOhm) were pulled from 1.5-mm borosilicate glass (Harvard Instruments, Kent, UK) using a DMZ Universal Puller (Zeitz Instruments, Munich, Germany) and filled with high-chloride internal solution (130 mM CsCl, 10 mM EGTA, 1 mM MgCl_2_, 10 mM HEPES, 2 mM ATP, 0.3 mM GTP, 10 mM phosphocreatine, and 1 mM CaCl_2_, pH adjusted to 7.3 with CsOH 295–300 mOsm; all chemicals from Sigma–Aldrich). The series resistance was compensated to values between 1.8 and 8 MOhm with a lag time of 10 μs.

5 mM QX-314 (Alomone Labs, Jerusalem, Israel) was added to the internal solution to eliminate postsynaptic sodium currents. Glutamatergic currents were blocked by addition of 40 μM DNQX and 50 μM D-AP-V (both from Tocris Bioscience, Bristol, UK) to the ACSF. In some recordings GABAergic currents were blocked by 20 μM SR 95531 (Tocris Bioscience). In other recordings, glycine currents were blocked by 500 nM strychnine (Sigma).

#### Electrical stimulation of inhibitory inputs

Inhibitory postsynaptic currents (IPSCs) were evoked by electrical stimulation in the vicinity of the MNTB principal neuron via an ACSF-filled glass pipette with tip resistance of 2–3 MOhm. The location and intensity of the stimulus were optimized to obtain the largest IPSCs. Stimuli were 100-μs-long square pulses of 1 to 90 V delivered with an STG 2004 computer-controlled four-channel stimulator (Multi Channel Systems, Reutlingen, Germany) and a stimulation isolation unit (Iso-flex, AMPI, Jerusalem, Israel). IPSCs were analyzed in IGOR Pro 6.21 (WaveMetrics), Clampfit 10.3.0.2 (Molecular Devices, Sunnyvale, CA) and Axograph X (Axograph.com).

## Results

### GlyT2-GFP transgenic mice reveal glycinergic neurons in auditory brainstem

The main goal of this study was to identify sources of glycinergic inhibition to principal neurons in the MNTB. In GlyT2-GFP mice, somata of glycinergic neurons are GFP+, as are axons in some cases. In auditory brainstem nuclei in a medio-ventral region of a P14 transgenic mouse, neurons of the MNTB, VNTB, LNTB, SPN, as well as a LSO subpopulation, known to be at least partially glycinergic, are GFP+ (Helfert et al., 1989; Figure 2A). VNTB and LNTB are recognized on the basis of their GFP expression and the GFP^−^ gap between them (Figures 2B,C).

### Retrograde tracer injections into MNTB reveal the ipsilateral VNTB as a putative source of glycinergic inhibition to MNTB

**Figure 3:**
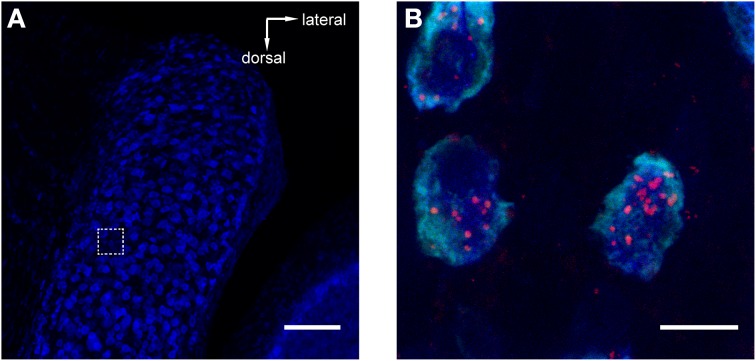
**Retrograde tracer injections into MNTB resulted in labeling of neurons in the antero-ventral cochlear nucleus (AVCN).** Red = TMR, blue = Nissl. **(A)** Image of AVCN in Nissl only to highlight the globular shapes of cells in the approximate location where globular bushy cells (GCBs) are expected. The dashed square indicates the area from which the magnification in **(B)** was imaged. The image is a maximum projection of a 80 μm stack. **(B)** Maximum projection computed from a sub-stack through the mid-sections of 3 labeled GBCs (spanning a total of 9 μm). Note the punctate labeling of the cell bodies. Scale bar for **(A)**: 100 μm, for **(B)**: 10 μm; 4.6× digital zoom.

To reveal potential sources of synaptic input to MNTB, we injected retrograde tracers into the MNTB of GlyT2-GFP mice. One known input nucleus is the contralateral AVCN, the home of globular bushy cells whose axons give rise to the calyx of Held(1893; Morest, 1968). As expected, we detected the tracer in globular bushy-like cells (on basis of shape and location; Webster and Trune, 1982; Kuwabara et al., 1991; Smith et al., 1991) of the AVCN (Figures 3A,B). To exclude the possibility that the tracer material was located in presynaptic terminals surrounding these somata, we analyzed single confocal sections and substacks consisting of only the center portions of the cell somata. We found red label clearly inside the center somata of neurons and several micrometers distant from the cell membranes, making it unlikely that it could represent presynaptic terminals surrounding these somata (Figure 3B). The results from this control experiment indicate that the tracer injection correctly targeted the MNTB suggesting successful uptake and transport to source neurons.

**Figure 4:**
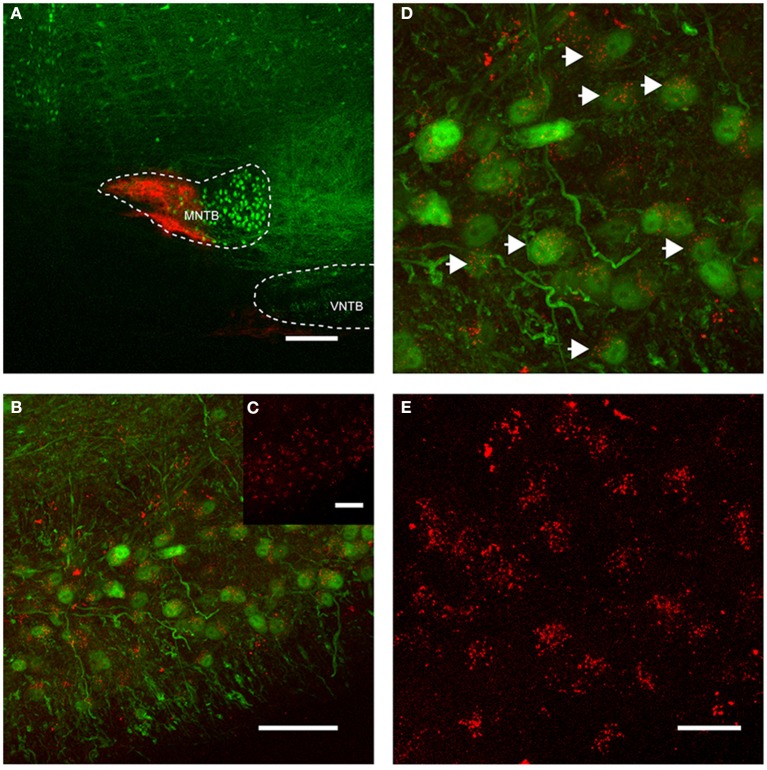
**Retrograde labeling of MNTB resulted in labeling of glycinergic neurons in the ipsilateral VNTB.** The image is from a case in which CTB was injected into the MNTB of a p88 GlyT2-GFP mouse. Glycinergic neurons expressing GFP are shown in green, the tracer is shown in red. **(A)** Shows the site of injection targeting mostly the medial half of the MNTB. **(B)** Depicts the resulting label in the ipsilateral VNTB [the inset **(C)** shows the red channel for this image only]. **(D)** Is a magnification of a portion of the image shown in **(B)**, with **(E)** showing the corresponding red channel only. The white arrows in **(D)** highlight double-labeled neurons. Images are maximum projections of confocal stacks (image depth: 60 μm). Scale bar for **(A)**: 200 μm, **(B,C)**: 50 μm, for **(D,E)**: 20 μm.

More importantly, we also found somata in the ipsilateral VNTB that were labeled in a similar fashion as the globular bushy cells described above (Figure 4). This CTB injection into the medial portion of the MNTB (Figure 4A) resulted in retrograde label in the VNTB, ipsilateral to the injection site (Figures 4B–E). These results raise the possibility that VNTB neurons send synaptic inputs to the ipsilateral MNTB, and that these inputs are glycinergic.

**Figure 5:**
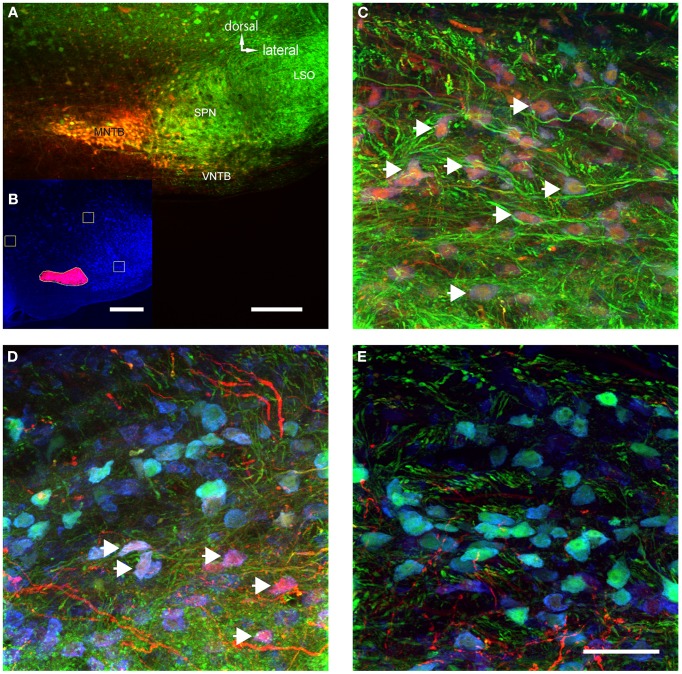
**The volume of TMR-injections into MNTB correlates with the number of labeled VNTB neurons. (A)** A brain stem image showing a TMR-injected (red) MNTB. **(B)** The result of a semi-automatic analysis of the same injection with our custom-built MATLAB algorithm, illustrating the method of quantifying the apparent extent of the tracer injection. **(C)** Shows a section of VNTB from a brain where the tracer injection into the ipsilateral MNTB was extensive (65% of MNTB volume). **(D)** represents a section of VNTB from a brain where the tracer injection into the ipsilateral MNTB was moderate (5.5% of MNTB volume). **(E)** Represents a section of VNTB from a brain where the tracer injection into the ipsilateral MNTB was minimal (1.5% of MNTB volume). **(A,C–E)** are maximum projections of confocal stacks (image depth: 80 μm), **(B)** is based on an epifluorescence microscopy image taken at low magnification (10×). Scale bar for **(A)**: 200 μm, for **(B)**: 500 μm and **(C–E)**: 50 μm.

For all injections, we verified qualitatively the accuracy of the injection. In addition, for 7/18 cases, we performed quantitative analysis of the extent of the injection for the robust, dense labeling provided by TMR injections (Figures 5A,B; see Methods). The high concentration of dye and associated high levels of fluorescence makes it difficult to determine labeled presynaptic terminals in the area of the injection (but see Wang et al., 2014). Further, since intrinsic collaterals within the MNTB may exist (Guinan and Li, 1990; Kuwabara and Zook, 1991), it was not possible to distinguish between MNTB principal cells that took up the dye as a result of a “direct hit” vs. retrograde dye transport from labeled terminals to cell somata of other principal neurons. Therefore, the quantitative analysis of injection sites refers to the *apparent* injection, not the *effective* injection.

MNTB extends longitudinally along the rostro-caudal axis and thus, no single injection could evenly label tissue along this entire axis. Not surprisingly, the extent of the *apparent* tracer injection varied between 3 and 65% (average 13.8 ± 8.6%). However, when only a single section at which the tracer injection was centered was analyzed, the tracer injection varied between 8 and 100% of the MNTB surface area of that section (average = 27 ± 13%). In a case where 99% of the MNTB area was injected (Figure 5A), a majority of neurons in the ipsilateral VNTB were labeled with the tracer substance (white arrows, Figure 5C).

The number of retrogradely labeled neurons in VNTB varied with the extent of the tracer injections into MNTB (Figures 5C–E). Overall, larger injection volumes yielded more labeled neurons in the ipsilateral VNTB.

### Anterograde tracer injections into VNTB reveal labeled presynaptic terminals in the ipsilateral MNTB

The results from the retrograde injections with two different dyes (CTB and TMR) presented so far suggest that glycinergic VNTB neurons might send projections to the ipsilateral MNTB. To further substantiate this concept, we performed anterograde tracer injections into the VNTB. Anterograde tracer is taken up by postsynaptic neurons and labels their axons and presynaptic terminals. In our particular case, anterograde injections into the VNTB would be expected to label glycinergic presynaptic terminals synapsing onto MNTB principal neurons.

**Figure 6:**
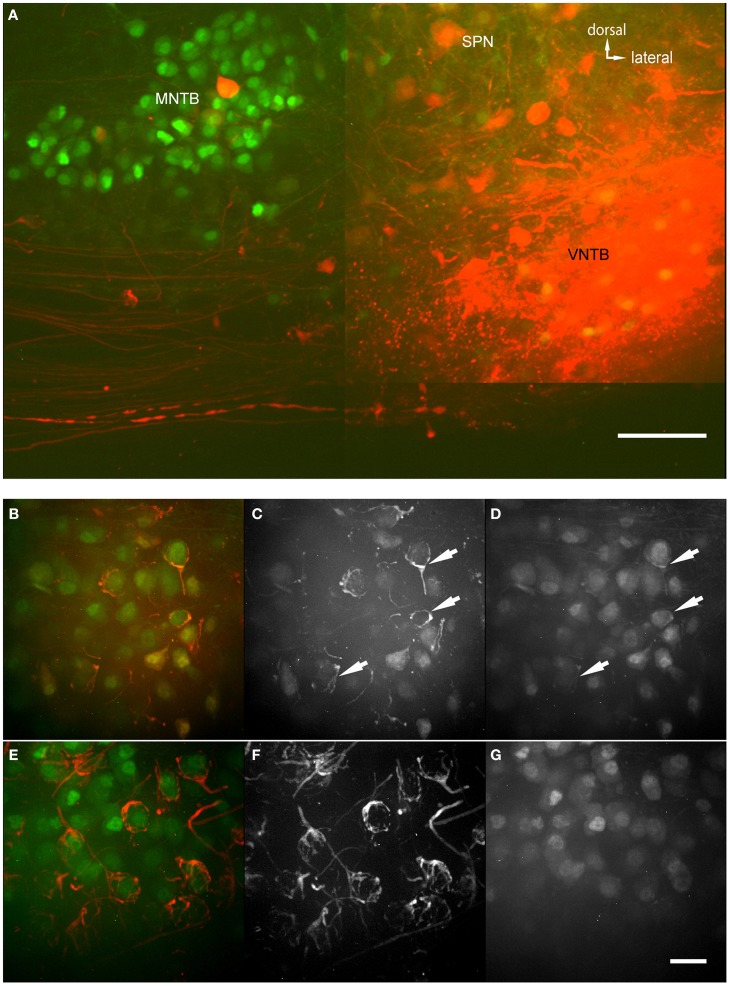
**Anterograde injections into VNTB reveal connections to MNTB neurons. (A)** Brain stem section of a case where TMR was injected into the VNTB of a p14 GlyT2-GFP mouse. The image shows the injection site within the VNTB, as well as the ipsilateral MNTB. A number of axons projects from the injection area to the ipsilateral MNTB and appears to terminate there. The image consists of a total of 4 tiled confocal stacks; the image depth of the original stack was 300 μm. Before imaging, the section was cleared using the Clear^T2^-protocol. **(B–D)** magnifications from the ipsilateral MNTB of a p14 brain that was cleared with the technique, showing a variety of labeled presynaptic elements. **(B)** Represents the overlay of both channels (red: TMR, green: GlyT2-GFP), **(C)** shows the red and **(D)** the green channel only. Double-labeled structures are highlighted with white arrows. **(E–G)** Since the majority of axons from AVCN to MNTB (innervating the calyces of Held) are passing through the injection area in the VNTB, typically some labeling of calyces of Held in the contralateral MNTB was observed as well. These were used as a control to compare to the labeled inhibitory endings in the ipsilateral MNTB. Note the structural differences between synapses labeled on the ipsilateral **(B–D)** and the contralateral side **(E–G)**, and the fact that at least some of the tracer-labeled ipsilateral structures are co-labeled with GFP, while the contralateral ones are not. Scale bar for **(A)**: 100 μm, for **(B–G)**: 50 μm.

The anterograde injections were performed into brainstem explants, similar to the retrograde injections, with two differences (Figure 6A): (1) The brain stem explant was cut differently and injected differently, and (2) blocks of tissue were cut into thicker sections and “cleared” before imaging (Clear^T2^ after Kuwajima et al., 2013). Clearing involves incubating the brain section in a solution that has a similar refractory index as the myelin to make it optically transparent. In cleared material, we were able to follow single projections from VNTB neurons to MNTB (Figure 6A). The injection site of the anterograde tracer is evident (bright saturated red spot, Figure 6A), as well as labeling in the ventral acoustic stria where a number of fiber bundles run through the VNTB. Importantly, the injection resulted in labeling projections from VNTB to MNTB, as well as associated glycinergic presynaptic terminals surrounding *ipsilateral* MNTB principal neurons (Figures 6B–D). Since the area of the VNTB also contains fibers of the acoustic stria which run e.g., from the cochlear nucleus to the contralateral MNTB, some of these passing fibers were also labeled by the injection (Figures 6E–G). In the *contralateral* MNTB, calyces of Held are innervated by labeled fibers passing through the contralateral VNTB. Even though some inhibitory inputs appear to look somewhat like calyces (e.g., Figure 6B), calyces of Held branch much more extensively and surround MNTB postsynaptic neurons more completely (Figures 6E,F). More importantly, at least some of the *ipsilaterally* tracer-labeled presynaptic elements are GFP+ (Figures 6B–D), suggesting that these ipsilaterally labeled structures are of glycinergic nature. By contrast, the *contralaterally* labeled calyces of Held are *not* GFP+ (Figures 6F,G), suggesting that these contralaterally labeled structures are *not* of glycinergic nature.

**Figure 7:**
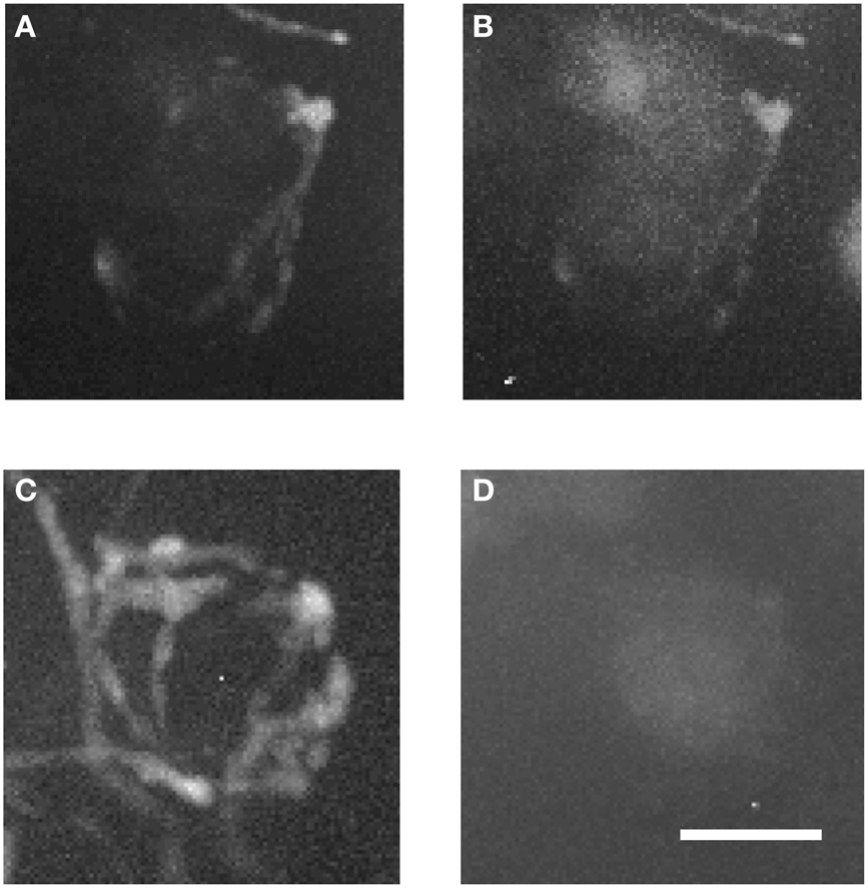
**Two magnified MNTB principal cells and their TMR-traced inputs. (A,B)** are showing a cell located ipsilaterally to the injected VNTB, **(C,D)** a contralateral one. Note the structural differences between the two input types and the fact that the ipsilateral terminal can be seen in the green channel (coding for GlyT2-GFP). Scale bar: 25 μm.

Figure 7 shows magnified examples of inputs to MNTB cells that arise from contralateral vs. ipsilateral nuclei which have significant structural differences. Firstly, the ipsilaterally labeled inputs branch much less than the contralaterally labeled inputs (Figure 7A vs. Figure 7C). Secondly, the ipsilaterally labeled structures are glycinergic while the contralaterally labeled structures are not (Figure 7B vs. Figure 7D). In total, we performed anterograde injections into 18 brainstem explants, and found labeled presynaptic terminals in ipsilateral MNTB in each case.

### Immunohistochemical labeling against GlyT2 revealed similarly shaped presynaptic terminals in MNTB

Interestingly, the presynaptic terminals shown in Figures 6B–D suggest that several presynptic boutons may be innervated by the same fiber, which runs along the target neuron and makes several connections. While this finding is consistent with physiological results (Florian Mayer, unpublished data), we wanted to further interrogate this innervation pattern with immuno-histochemistry against neuronal glycine transporter 2 (GlyT2). Thus, if the putative presynaptic terminals shown in Figures 6B,C are indeed presynaptic terminals and not simply axons or dendrites of glycinergic neurons, they should express GlyT2 protein.

**Figure 8:**
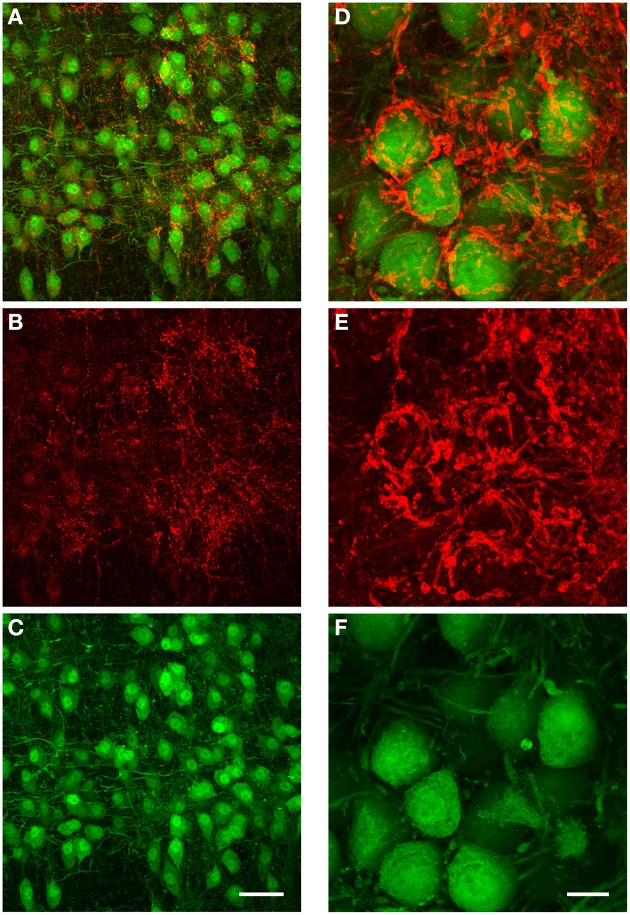
**Immunohistochemical labeling against GlyT2 protein reveals complex inhibitory synaptic structures surrounding MNTB principal neurons.** All images are maximum projections of a confocal stack [image depth for **(A–C)**: 40 μm, for **(D–F)**: 20 μm]. The overlay of both channels is displayed in **(A)**. The antibody labeling against GlyT2 is shown in red **(B)** and the GFP-expression under the GlyT2-promoter in green **(C)**. Note that many of the presynaptic terminals labeled by the GlyT2 antibody are highly complex in their structure, and similar to the inhibitory synaptic inputs labeled with the anterograde TMR-injections into the ipsilateral VNTB shown in Figures 6B–D. **(D–F)**: Higher magnification image (3× digital zoom) taken in the MNTB of a different GlyT2-GFP mouse. Scale bar for **(A–C)**: 50 μm, for **(D–F)**: 10 μm. The age of the animal was p59.

We performed immunohistochemistry against GlyT2 protein in 5 brains obtained from both GlyT2-GFP negative (= wild type) and GlyT2-GFP mice (2 wild type and 3 GlyT2-GFP mice; data for wild type mice not shown). The distribution of GlyT2 protein in the boutons near MNTB principal neurons suggests that these boutons are indeed presynaptic terminals (Figure 8). The dense and complex structure of many of these terminals (Figures 8A,D) is reminiscent of the presynaptic elements labeled in our anterograde injections (Figures 6B,C), further supporting the notion that these structures might be glycinergic presynaptic terminals, several of which are innervated by the same fiber.

### Parallel developmental changes from GABA to glycine in MNTB and VNTB

**Figure 9:**
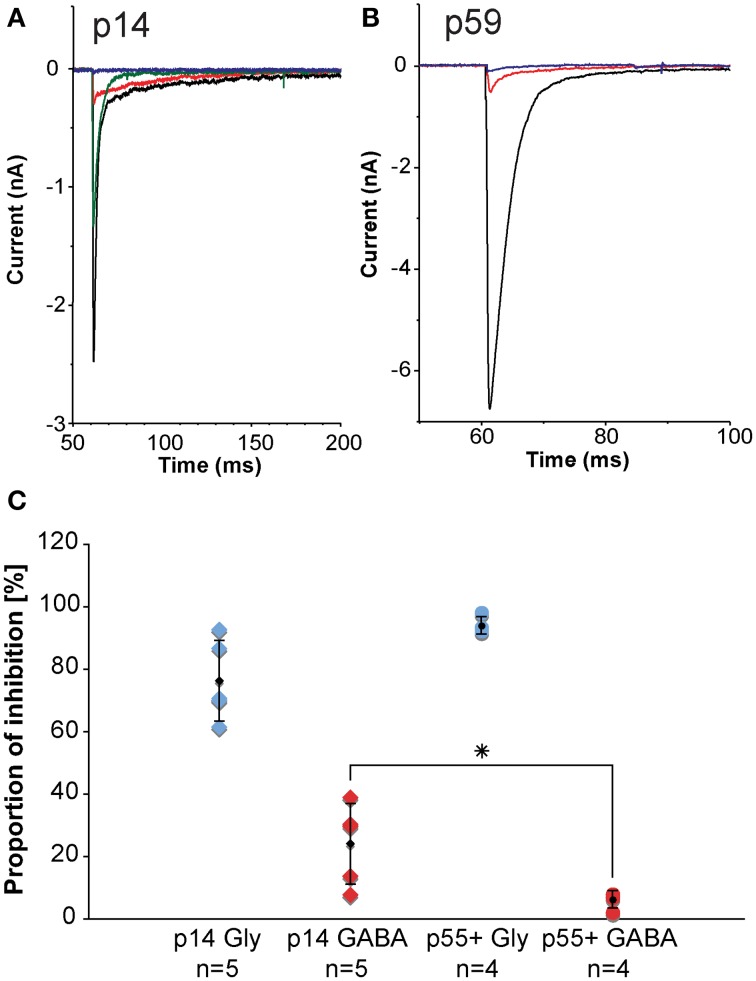
***In-vitro* electrophysiology in the mouse reveals that the GABAergic contribution to the total inhibition at MNTB principal neurons declines with age. (A,B)** are representative examples of pharmacologically isolated inhibitory currents observed in a p14 **(A)** and a p59 **(B)** mouse and measured while inhibitory synapses were electrically stimulated in the vicinity of the MNTB principal neuron. The black trace is the total inhibitory current following electrical stimulation, the red trace shows the residual GABAergic current after blocking the glycine component with strychnine, the blue trace is the complete block after an additional wash-in of SR-95531 (gabazine). The green trace shows a partial recovery after a successful washout of both drugs. **(C)** Normalized current amplitudes for glycine currents suggest a developmental decline of the GABAergic contribution to the total inhibition. Blue diamonds: normalized glycinergic currents measured from p14 animals; blue dots: normalized glycinergic currents measured from p55+ animals; red diamonds: normalized GABA currents measured from p14 animals; red dots: normalized GABA currents measured from for p55+ animals; black symbols: averages with error bars (SD). The GABA component declines significantly with age from almost 25% of total inhibitory current at postnatal day 14 down to about 6% in adult animals, whereas the glycine component does not experience statistically significant changes. All excitatory currents were blocked during these experiments with the glutamate receptor blockers DNQX and AP-V. All recordings were done near physiological temperature (35–37°C), which significantly speeds up channel kinetics (Leao et al., 2005). ^*^The *p*-value is 0.032 (t-test).

Inhibitory inputs to MNTB undergo developmental changes. During early postnatal ages, inhibitory inputs to MNTB have both a GABAergic component and a glycinergic component. During postnatal development, the GABAergic contribution gets progressively smaller such that during later developmental stages, the inhibition to MNTB neurons is almost exclusively glycinergic. We assessed the sizes of the GABAergic and glycinergic components by using pharmacological blockers (Figure 9). From the amplitudes of the mixed and purely GABAergic/glycinergic currents, ratios were calculated. Figure 9C shows ratios of GABAergic and glycinergic currents at p14 (left group of data points, *n* = 5), and p55+ (right group of data points, *n* = 4), suggesting that at the younger age, 24% of the total inhibitory current is GABAergic. By age p55+, the contribution of GABA has dropped to 6%.

**Figure 10:**
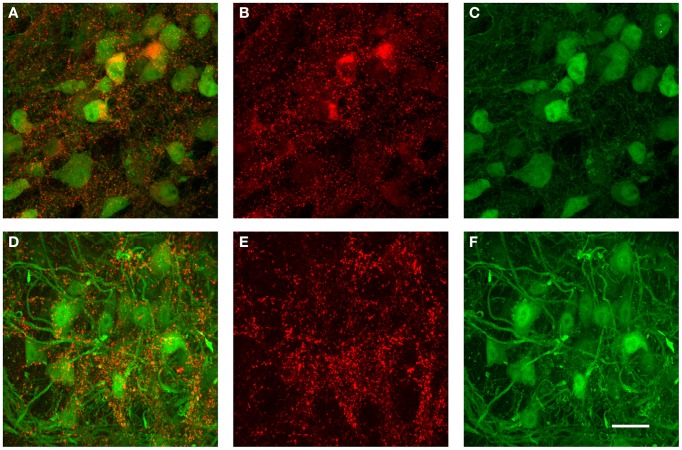
**Examples of immunohistochemical label against GAD67 in the VNTB of GlyT2-GFP mice. (A–C)** show a representative anti-GAD67 labeling pattern in the VNTB of a p14 mouse, with **(A)** being the overlay of both channels, **(B)** the antibody labeling in red and **(C)** the GlyT2-GFP expression in green. Images shown in **(D–F)** show the same labeling in an adult animal aged p70 (color coding is the same as in **(A–C)**]. Note that at p14, many glycinergic neurons are co-labeled with GAD67, while almost none of the glycinergic neurons seen at the older age are co-labeled against GAD67. All images are maximum projections of confocal stacks. Scale bar: 20 μm.

The data from the tracer injections shown above suggest that inhibitory projections from VNTB to MNTB may represent a major source of inhibition to MNTB. If that is the case, but inhibitory inputs to MNTB undergo a developmental change during which the contribution of GABA to the total inhibition decreases (Figure 9), one might postulate that VNTB neurons undergo a developmental switch from GABA to glycine that mirrors the switch observed at their target neurons in the MNTB. We addressed this question with immunohistochemical labeling against glutamic acid decarboxylase isoform 67 (GAD67) in both p14 mice and in p50+ mice (Figure 10). GAD67 protein catalyzes the decarboxylation of glutamate to GABA and CO_2_ and thus is a marker of GABAergic neurons. In the younger age group, substantial amounts of GAD67 labeling can be observed within VNTB principal neurons (Figures 10A–C), while in the older age group, much less label can be observed within the postsynaptic neurons (Figures 10D–F). Note that in both age groups there appears to be some small punctate GAD67 label outside of principal neurons. It is unclear whether these structures represent GABAergic synapses or other structures. However and importantly, GAD67 label inside the VNTB principal cells is highly reduced in the older age group.

**Figure 11:**
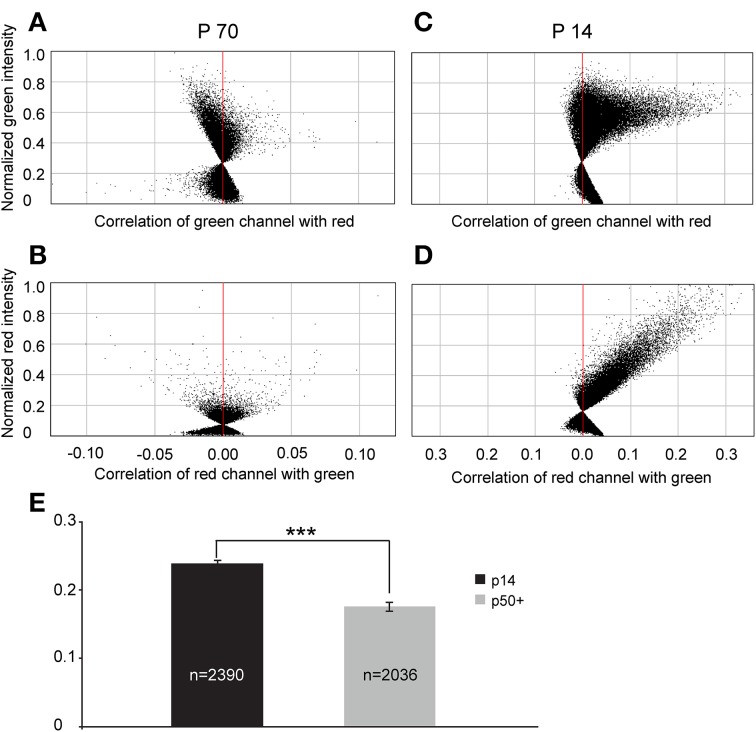
**Intrasomatic labeling against GAD67 in glycinergic VNTB neurons declines with age. (A–D)** show pixel data from two single glycinergic VNTB neurons. Single glycinergic VNTB neurons were selected in a region of interest and analyzed for co-localization of signals in both channels. **(A,B)** display the normalized intensity correlation values of pixels in a p70 animal for the green and the red channel, respectively. Most of the values do not exceed the intensity correlation quotient (ICQ) range of −0.05 to 0.05 and are mostly distributed around the midline, indicating a largely uncorrelated or random distribution of overlapping green and red pixels (the resulting total ICQ was −0.001 in this case). By contrast, **(C,D)** show a strongly positive correlation of signals in the green and the red channels, respectively (with a resulting total ICQ value of 0.38 for this specific neuron). **(E)** Averaged total ICQ values for 2390 glycinergic VNTB neurons in p14 animals (black) and 2036 glycinergic VNTB neurons in p50+ (p59–70; gray) animals, respectively. ^***^p < 0.001 (Mann-Whitney rank-sum test).

To quantify this reduction, we performed Li's intensity correlation analysis to assess the extent of colocalization (Li et al., 2004) between the GFP signal and GAD immunoreactivity (*n* = 11 of GlyT2-GFP brains). This intensity correlation analysis revealed a significant degree of colocalization at p14. However, at p50+, we calculated overall lower ICQ values between the GFP and GAD signals (see representative examples in Figures 11A–D and ICQ averages in Figure 11E). These results, together with the physiological recordings from MNTB principal neurons (Figure 9) suggest a parallel developmental switch from GABA to glycine in VNTB principal neurons (from which inhibitory inputs to MNTB originate) and inhibitory inputs to MNTB (where projections from VNTB terminate), providing further evidence for the idea that VNTB is a major source of inhibitory inputs to MNTB.

## Discussion

The main conclusion from our study is that MNTB neurons in the mouse receive prominent glycinergic inputs from neurons in the VNTB. These neurons send projections to MNTB, which end in several synaptic boutons located on the somata of MNTB principal neurons. This conclusion is supported by several lines of evidence: retrograde tracer injections into MNTB label the somata of VNTB neurons intracellularly, anterograde tracer injections into VNTB label presynaptic glycinergic boutons on MNTB principal neurons, and the developmental switch of VNTB neurons from mixed GABAergic/glycinergic to almost exclusively glycinergic is matched by a parallel developmental switch of incoming inhibition to MNTB neurons from mixed GABAergic/glycinergic to almost exclusively glycinergic.

The method of injecting brain stem explants with tracer in a dish and subsequently incubating the tissue for a few hours *in-vitro* (Burger et al., 2005; Ford et al., 2009) greatly facilitated this study. Since both MNTB and VNTB are very close to the ventral surface of the brain stem, and are almost “surface structures” from a ventral view, tracer injections could be performed with great accuracy under visual guidance. Cell death during the relatively brief incubation time was very limited. The method of clearing brain tissue with the Clear^T2^ method (Kuwajima et al., 2013) worked relatively well in auditory brain stem sections, especially in those from younger animals. Fibers in the auditory brain stem myelinate extensively (Ryugo et al., 2006), which greatly impairs imaging quality both for physiological and anatomical studies. The brain clearing allowed us to prepare relatively thick sections of 200–500 micrometers, in which intact connections between VNTB and MNTB could be visualized. Nevertheless, in highly myelinated adult brain stem, this technique resulted in only incomplete clearing.

The results from the retrograde tracing studies alone, though conducted with two different tracers (CTB and TMR) would not be sufficient to demonstrate inhibitory connections between VNTB and MNTB because the VNTB also projects to *contralateral* SOC nuclei, such as the LSO (Spangler et al., 1985; Warr and Beck, 1996), the LNTB and DLPO (Warr and Beck, 1996; Thompson and Schofield, 2000), with associated fibers running close to or even through the ipsilateral MNTB. We therefore also performed anterograde injections into the VNTB, combined with subsequent brain clearing. In these experiments we were able to observe direct axonal connections running from VNTB to MNTB, as well as labeled presynaptic terminals within the MNTB. Though we cannot completely exclude the possibility of labeling potential *ipsilaterally* projecting calyces and/or collaterals of calyceal inputs that project to VNTB (Kuwabara et al., 1991), we think this possibility is unlikely for two reasons: First, a comparison to contralaterally labeled calyces of Held in the same brain slices revealed that the morphology of true calyces of Held is significantly different from the morphology of the putative inhibitory inputs. Second, contralaterally labeled calyces did not co-label for the GlyT2-GFP marker, but ipsilaterally labeled putative inhibitory inputs did. Although our data present evidence for a glycinergic projection from VNTB to MNTB, we cannot rule out other potential sources of inhibition to MNTB. Especially, intrinsic connections within MNTB have been previously suggested as a second source of inhibition to MNTB neurons (Guinan and Li, 1990; Kuwabara and Zook, 1991). Our data does not address this possibility.

There are several pitfalls when using tracer injections to study connections between different brain nuclei. One of them is the difference between the *apparent* and the *effective* size of an injection site. One proven method to determine the effective size of an injection area consists of counting labeled neurons at the site of injection (e.g., Wang et al., 2014). There are several reasons, why we did not use this method in our study, and restricted ourselves to delineating only the *apparent* site of injection in the MNTB. Firstly, in our experiments the differences between the apparent and the effective sites of injection were presumably relatively small due to the fact that the brain stem explants were kept alive for relatively short amounts of time (1–4 h). In contrast, fluorescent dextran-based tracer labeling fades over days and weeks (Novikova et al., 1997; also see Köbbert et al., 2000 for a detailed review and comparison of neuroanatomical tracing techniques). More importantly, since TMR served as a *retrograde* tracer, the most accurate method to determine the effective size of injection would be counting the inhibitory synaptic inputs around MNTB cells. Determining labeled presynaptic terminals in the immensely bright area of the injection site proved to be impossible. Alternatively, assessing the effective injection site by labeled *post*synaptic cells would be less accurate because presynaptic terminals take up dye with different mechanisms as postsynaptic neurons do, resulting in inaccurate quantifications of the effective injection site. Moreover, a number of studies suggested intrinsic inhibitory connections within the MNTB (Guinan and Li, 1990; Kuwabara and Zook, 1991), making it impossible to distinguish between a postsynaptic neuron labeled by a “direct hit” and one that was labeled intrasomatically due to retrograde transport through recurrent collaterals.

Another potential pitfall to consider would include retrograde labeling of MNTB neurons projecting to VNTB (Kuwabara and Zook, 1991). If this was the case, observed label within the MNTB would have resulted from inhibitory terminals formed by (other) MNTB neurons that took up tracer material retrogradely through an axonal branch extending to VNTB, and subsequently transported the material from there to a collateral projecting to neighboring MNTB neurons. We think this possibility is unlikely because of the intensity differences of the fluorescent label between the labeled terminals surrounding MNTB cells and retrogradely labeled MNTB cell somata. While we could observe a subset of MNTB cells that were labeled retrogradely, their staining intensities were in most cases much lower than the staining intensities of the labeled inhibitory terminals surrounding principal cells.

GlyT2 has been shown to be a reliable marker of glycinergic neurons (Poyatos et al., 1997; Zeilhofer et al., 2005). Moreover, the GFP-expression pattern seen in the SOC of the GlyT2-GFP mouse line used here is consistent with observations made by Zeilhofer et al. (2005), as well as immunohistochemical and tracing studies from other investigators in different rodent species (MNTB, LNTB, MVPO/VNTB: Peyret et al., 1987; Helfert et al., 1989; SPN: Helfert et al., 1989; Saint Marie and Baker, 1990). Thus, the GFP expression in this mouse line is specific and reliably shows the (sub)populations of glycinergic cells in the auditory brainstem nuclei despite some variability in the *level* of GFP-expression between different nuclei and individual animals.

The nomenclature and the outlines of some auditory brainstem nuclei in rodents, especially the periolivary nuclei have been somewhat inconsistent. In the VNTB literature, at least six different names in three closely related rodent species (mouse, rat, and deermouse) have been used to describe the heterogeneous group of cells located ventrolaterally to the location of MNTB: VNTB appears to be the most commonly used term (see Thompson and Schofield, 2000 for a review). Other terms include IPN (internal parolivary nucleus in the deermouse; Ross, 1962) MPVO (medial preolivary nucleus in the deermouse, Ross, 1969), MVPO (e.g., Paxinos and Franklin Mouse Brain Atlas), VTN (ventral trapezoid nucleus in the rat; e.g., Brown and Howlett, 1972; see Ollo and Schwartz, 1979 for a review). Other investigators have subdivided the nucleus into VNTB and RPO (rostral periolivary region), based on morphology and basic electrophysiology in the rat (Robertson, 1996).

Whereas our study did not attempt to resolve these questions or define the outlines of the VNTB *in its entirety*, we did define the glycinergic subpopulation of VNTB principal cells that are GFP+ in the transgenic mouse line. We found that it closely matches the outlines defined by other authors who previously studied the anatomy of the mouse SOC more intensely (see Ollo and Schwartz, 1979; Franklin and Paxinos, 2008). Based on the patterns of GFP-expression and the morphology of GFP-positive, i.e., glycinergic cells, we were able to distinguish between the VNTB and other neighboring periolivary regions like the SPN and the LNTB with relative ease.

While connections between MNTB and VNTB have been suggested before (Kuwabara et al., 1991; Thompson and Schofield, 2000), the extent and the glycinergic nature of these projections have not been shown. VNTB contains several neuronal types, some of which are glycinergic (Helfert et al., 1989), while others are cholinergic or GABAergic (Helfert et al., 1989; Yao and Godfrey, 1998; Gómez-Nieto et al., 2008). We focused on the glycinergic subpopulation, because previous studies suggested that inhibitory inputs to MNTB principal cells are largely glycinergic (at least in the adult rodent; Awatramani et al., 2005). Moreover, glycinergic inhibition from VNTB and LNTB might play important roles in sound localization processing. For example, Roberts et al. (2013) showed that inhibition to the MSO (which originates from MNTB and LNTB; Thompson and Schofield, 2000) can arrive even *before* the excitation. This study and others are contributing to the idea that inhibition is playing a crucial, yet still not completely understood role in the auditory brain stem.

Lastly, Warr and Beck (1996) looked specifically at efferents of the VNTB and suggested that a projection from VNTB to MNTB “… does not appear to exist, at least in the rat, for VNTB neurons situated some distance away from the MNTB…” Our results are inconsistent with those findings from the rat, but support the findings of Kuwabara et al. (1991) in two other rodent species, the gerbil and the mouse, hinting at possible species-related differences in the intrinsic projection patterns within the SOC. Unfortunately, our anterograde injections into the VNTB could not discriminate between potential differences in the projection pattern between the medial and the lateral subpopulations of glycinergic VNTB neurons, which should be addressed in future studies (e.g., by using two different tracer colors for injections into the medial and the lateral portions of the VNTB).

Our data also confirm the idea that inhibitory inputs to MNTB are of auditory brainstem nature. This has been hypothesized by previous *in-vitro* studies due to the large synaptic amplitudes and fast decays produced by inhibitory synapses (Awatramani et al., 2004), as well as their ability to follow prolonged trains of ongoing stimulation. Although the amplitudes of the inhibitory currents do decrease with prolonged stimulation, the same has been shown by Hermann et al. (2007) for the excitation. Pharmacological manipulations performed *in-vivo*, however, have already seen evidence of the auditory nature of inhibitory inputs to MNTB neurons (Green and Sanes, 2005; Kopp-Scheinpflug et al., 2008; Tolnai et al., 2008). Since VNTB neurons receive excitatory input directly from the ventral cochlear nucleus on the contralateral side (Warr, 1972) their firing pattern most likely codes for relatively unprocessed auditory information, such as simple activity patterns and standard tuning curves.

We observed a developmental decrease in the contribution of GABA to the total inhibition innervating MNTB, as well as a parallel developmental decrease in GAD67 signal in glycinergic VNTB principal neurons. While the latter observation is novel, the first one is not. A similar decrease in the GABAergic contribution to total inhibition at MNTB neurons has been observed previously in the rat (Awatramani et al., 2005), suggesting that this decrease is a general mammalian phenomenon.

Both the anterograde tracer injections into VNTB and the GlyT2 immunolabels in MNTB revealed afferent fibers branching and terminating in several large synaptic boutons along MNTB principal cell somata. This innervation pattern suggests first of all that relatively few inhibitory synapses terminate on MNTB neurons and second of all that these boutons are connected to even fewer fibers. This result is consistent with our recent physiological data in which glycinergic input fibers were electrically stimulated in the vicinity of MNTB neurons in brain slices (Mayer et al., under review).

In summary, our data combining retrograde and anterograde tracing techniques, as well as demonstrating a simultaneous switch from GABA/glycine to mostly glycine in both the synaptic inputs to MNTB and the somata of glycinergic cells located in the VNTB, present a cumulative body of evidence suggesting that the glycinergic subset of VNTB neurons sends axonal connections to the MNTB, making the VNTB at least one major source of inhibition projecting to MNTB.

### Conflict of interest statement

The authors declare that the research was conducted in the absence of any commercial or financial relationships that could be construed as a potential conflict of interest.
